# Résultats du remplacement valvulaire chez les patients porteurs de rétrécissement valvulaire aortique avec dysfonction ventriculaire gauche sévère

**DOI:** 10.11604/pamj.2018.29.79.10991

**Published:** 2018-01-26

**Authors:** Abderrahmane Bakkali, Imad Jaabari, Claude Koulekey Dadji, Rochde Sayah, Mohamed Laaroussi

**Affiliations:** 1 Service de Chirurgie Cardiovasculaire « A », Hôpital Ibn Sina, Faculté de Médecine et de Pharmacie d'Agadir, Université Ibn Zohr, Agadir, Maroc; 2 Service de Chirurgie Cardiovasculaire « A », Hôpital Ibn Sina, Faculté de Médecine et de Pharmacie de Rabat, Université Mohamed V Souissi, Rabat, Maroc

**Keywords:** Rétrécissement aortique serré, remplacement valvulaire aortique, dysfonction ventriculaire gauche, Tight aortic stenosis, aortic valve replacement, left ventricular dysfunction

## Abstract

Le risque opératoire du remplacement valvulaire aortique(RVA) pour rétrécissement aortique (Rao) serré avec dysfonction ventriculaire gauche sévère demeure élevé. Plusieurs facteurs de risques de mortalité postopératoire sont décrits mais la plupart des séries rapportées sont hétérogènes. L'objectif principal de ce travail était d'étudier les résultats postopératoires du RVA chez les patients présentant un Rao serré isolé avec dysfonction ventriculaire gauche sévère et d'identifier les facteurs prédictifs de la mortalité hospitalière. Il s'agit d'une étude rétrospective qui a intéressé 46 patients porteurs de Rao serré avec dysfonction sévère du ventricule gauche et qui ont bénéficié d'un RVA. L'âge moyen était de 59±12,70 ans. 69,6% des patients étaient en classe III ou IV de la NYHA. La FE moyenne était de 32,3 ± 5,3 %, et l'EuroScore moyen était 12,20 ± 8,70. La mortalité hospitalière était de 15,20 %. La morbidité a été marquée essentiellement par le bas débit dans 35 % des cas. En analyse multivariée, la régression logistique a identifié l'insuffisance rénale (OR = 11,94, IC [2,65-72,22], p = 0,03) et l'insuffisance cardiaque congestive (OR = 25,33, IC [3,43-194,74], p = 0,009) comme étant liées au risque de mortalité hospitalière. Trente neuf patients survivants ont été suivis sur une durée moyenne de 59,6 ± 21mois. La mortalité tardive était de 5% .Le statut fonctionnel s'est nettement amélioré. La FE a augmenté, en moyenne, de 5,5 unités en période postopératoire précoce et de 18 unités en postopératoire tardive. A long terme les diamètres télédiastole et télésystole ont régressé en moyenne de 8 et 9mm respectivement. Les résultats du RVA sur RAo serré avec dysfonction ventriculaire gauche sévère sont satisfaisants. L'insuffisance cardiaque congestive et l'insuffisance rénale préopératoires constituent les principaux facteurs de risque de mortalité hospitalière. L'évolution tardive est marquée par une régression des diamètres télésystolique et télédiastolique du ventricule gauche avec amélioration de la FE et du statut fonctionnel.

## Abstract (en)

The operative risk of aortic valve replacement (AVR) due to tight aortic stenosis (AS) associated with severe left ventricular dysfunction is high. Several risk factors for postoperative mortality have been described, but most of the reported case series are heterogeneous. This study aimed to analyze the postoperative results of AVR in patients with isolated tight AS associated with severe left ventricular dysfunction and to identify predictive factors of in-hospital mortality. We conducted a retrospective study of 46 patients with tight AS associated with severe left ventricular dysfunction who had undergone AVR. The average age was 59±12.70 years. 69.6% of patients were in NYHA Class III or IV. Mean EF was 32.3 ± 5.3%, and mean EuroScore was 12.20 ± 8.70. In-hospital mortality accounted for 15.20%. Morbidity was mainly marked by low cardiac output in 35% of cases. Multivariate logistic regression analysis showed that renal insufficiency (OR= 11.94, CI [2.65-72.22], p= 0.03) and congestive cardiac failure (OR= 25.33, CI [3.43-194.74], p= 0.009) were related to the risk of in-hospital mortality. Thirty-nine surviving patients were followed up for an average of 59.6± 21 months. Late mortality accounted for 5%. The functional status had significantly improved. EF increased, on average, by 5.5 units in early postoperative period and by 18 units in late postoperative period. In the long term, end-diastolic and end-systolic diameters were reduced by an average of 8 and 9 mm, respectively. The results of AVR due to tight AS associated with severe left ventricular dysfunction are satisfactory. Congestive heart failure and preoperative renal failure are the main risk factors for in-hospital mortality. Patient’s outcome is marked by reduction in end-diastolic and end-systolic diameters of the left ventricle with improvement of the EF and of their functional status.

## Introduction

À l´heure actuelle, la chirurgie est le gold standard en matière du traitement des sténoses aortiques sévères [1]. Cependant, le risque chirurgical est augmenté lorsque la dysfonction ventriculaire gauche est présente [2]. La littérature rapporte plusieurs facteurs de risques liés à la mortalité postopératoire mais la plupart des séries publiées sont hétérogènes incluant des patients avec d'autres lésions associées, en particuliers des lésions coronaires ou valvulaires autres que le rétrécissement aortique. L'objectif principal de ce travail était d'étudier les résultats postopératoires du remplacement valvulaire aortique chez les patients présentant un rétrécissement aortique serré isolé avec dysfonction ventriculaire gauche sévère et d'identifier les facteurs prédictifs de la mortalité hospitalière. L'objectif secondaire était d'étudier l'évolution de la fonction ventriculaire gauche et son impact sur le statut fonctionnel de ces patients.

## Méthodes

**Patients:** Il s'agit d'une étude transversale rétrospective étalée, sur une période allant de janvier 2000 à janvier 2016, qui a intéressé 46 patients porteurs de rétrécissement aortique serré(RAo) avec dysfonction sévère du ventricule gauche (VG) et qui ont bénéficié d'un remplacement valvulaire aortique (RVA) dans notre département.

**Les critères d'inclusion étaient:** 1) Rétrécissement aortique serré (surface aortique < 1 cm^2^ ou surface aortique indexée < 0,6 cm^2^/m^2^); 2) fraction d'éjection (FE) < 40%; 3) les patients régulièrement suivis à notre consultation ou à celle de leurs médecins traitants.

**Les critères d'exclusion étaient:** 1) L'existence d'une fuite aortique > grade I; 2) la présence d'une autre valvulopathie associée ayant nécessité une correction chirurgicale; 3) la présence d'une coronaropathie au stade chirurgical ou d'antécédent d'infarctus du myocarde; 4) les patients opérés d'un rétrécissement aortique serré avec dysfonction ventriculaire gauche et qui sont perdu de vue.

**Techniques opératoires:** Le remplacement valvulaire aortique a été effectué, via une sternotomie médiane, sous circulation extracorporelle (CEC) menée en hypothermie modérée. Jusqu'à l'an 2002, la protection myocardique a été assurée par une cardioplégie cristalloïde froide intermittente (Saint Thomas). Après cette date, une cardioplégie froide au sang a été utilisée.

**Méthodes:** Les données cliniques, paracliniques et opératoires ont été recueillies des dossiers médicaux des patients. Le contrôle postopératoire a été effectué soit par les cardiologues du service en convoquant les patients, soit par les cardiologues traitants qui nous ont transmis les données de leurs examens. Le contrôle a porté sur le statut fonctionnel selon la classe de NYHA et sur les données des examens échocardiogrphiques. Les données échocardiogrphiques répondaient aux recommandations de la société européenne d'échocardiographie [3] et celles de la société américaine d'échocardiographie [4]. Les dimensions du VG ont été obtenues par le mode TM et bidimensionnel (BD), la surface Aortique a été calculée par la méthode d'équation de continuité et le gradient de pression transvalvulaire par la méthode d'équation de Bernoulli. La fraction d'éjection du VG a été évaluée par la méthode de Simpson. Tous les patients ont bénéficié d'une coronarographie sauf une patiente opérée en urgence extrême.

**Définitions:** La période postopératoire précoce est définie par les 6 premiers mois suivant la chirurgie. Le post opératoire tardif a été considéré comme une période de suivi supérieure à un an. Le bas gradient aortique est défini par un gradient trans-aortique < 40 mmHg. La mortalité hospitalière est définie par tout décès survenu dans les 30 jours suivant la chirurgie. Le bas débit cardiaque est défini par un index cardiaque ≤2l/min/m ^2^ ou lors d'un besoin aux catécholamines > 6 gamma/Kg/min.

**Analyse statistique:** L'analyse statistique a été faite par le logiciel « Statistical Package for the Social Sciences » (SPSS version 11.5, Chicago, Illinois, USA). La distribution des variables quantitatives a été testée par le test de Kolmogorov-Smirnov. Les variables quantitatives de distribution gaussienne sont exprimées en moyenne ± écart type, les non gaussiennes en médiane et intervalle interquartile (IQ) et les variables qualitatives en fréquence et pourcentage. La comparaison des données a été faite par, le test t de Student pour les variables de distribution gaussienne, le test de Mann-Whitney pour les variables de distribution non gaussienne et le test de Ki2 ou le test exact de Fisher pour les variables qualitatives. La comparaison des groupes appariés a été faite par analyse de variance sur mesures répétées pour les variables de distribution normale et par le test de Friedman pour les variables de distribution asymétrique. Les facteurs de risque de mortalité ont été étudiés par l'analyse de régression logistique et présentés par l'Odds-ratio (OR) avec un intervalle de confiance de 95% L'estimation de la courbe de survie a fait appel au taux de survie réactualisé à chaque temps de survenue d'un événement selon la méthode de Kaplan-Meier. Une valeur de p<0,05 a été fixé comme seuil de significativité.

## Résultats

Sur une période de 16 ans nous avons pu recueillir 46 patients porteurs de RAo serré avec dysfonction sévère du ventricule gauche (VG) et qui ont bénéficié d'un RVA. L'âge moyen était de 59±12,70 ans avec une nette prédominance masculine. Tous les patients ont été symptomatiques, 69,6% d'entre eux étaient en classe III ou IV de la NYHA. La FE moyenne était de 32,3 ± 5,3 % et l'EuroScore moyen était 12,20 ± 8,70. Seuls 3 malades ont bénéficié d'une étude de la réserve contractile, par échocardiographie sous faible dose de dobutamine, qui s'est révélée positif dans les 3 cas. Les caractéristiques générales de nos patients sont représentées dans le Tableau 1. Trente neuf prothèses mécaniques et 7 bioprothèses, dont le diamètre moyen était de 21,8 ± 1,6 mm, ont été implantées. Le temps de Clampage Aortique moyen était 70,5 ± 21,7 mn avec une médiane du temps totale de CEC de 91,5 mn (IQ [80,7-129,5]). Le recours aux drogues inotropes a été nécessaire dans 91,3% des cas. La médiane du délai d'extubation était de 8,5 heures et celle de la durée de séjour en réanimation de 48 heures. La mortalité hospitalière était de 15,20 %. La morbidité a été marquée essentiellement par un bas débit dans 35% des cas. Les données opératoires et postopératoires précoces sont rapportées dans le Tableau 2. L'étude de la régression logistique en analyse univariée a permis de révéler la classe fonctionnelle, l'insuffisance rénale, l'insuffisance cardiaque congestive préopératoire, la FE et le temps de la CEC comme facteurs liés au risque de mortalité postopératoire précoce. En effet le risque de mortalité postopératoire est multiplié par 8,20 lorsque la NYHA augmente d'une classe. La présence d'une insuffisance cardiaque congestive ou d'une insuffisance rénale, en préopératoire, multiplient le risque opératoire, respectivement, par 24,66 et 11,66. De même, l'allongement du temps de CEC d'une minute multiplie le risque opératoire par 1,02 (OR= 1,02, IC [1,00-1,04], p= 0,02), alors que l'augmentation de la FE préopératoire d'une unité divise ce risque par 1,16 (OR= 0,86, IC [0,77-0,96], p= 0,01). En analyse multivariée, seuls l'insuffisance rénale et l'insuffisance cardiaque congestive paraissent liées au risque de mortalité précoce (Tableau 3).

**Tableau 1:** Caractéristiques générales de la population étudiée

Variables	n = 46
Age^1^ (années)	59 ± 12,7
Sexe^3^ M/F	37/9 (80,4 %)
Surface corporelle^2^	1,7 [1,67- 1,85]
NYHA^3^	
-II	14 (30,4%)
-III	20 (43,5%)
-IV	12 (26,1%)
Angor^3^	18 (39%)
Syncope^3^	4 (8,7%)
ICC^3^	6 (13%)
Nature de la valvulopathie^3^	
-Dégénérative	32 (69,6%)
-Rhumatismale	12 (21,1%)
-Congénitale	2 (4,3%)
**Comorbidités^3^**	
-HTA	17 (37%)
-Diabète	5 (11%)
-IR	8 (17,4%)
-AVCI	1 (2,2%)
RCT^2^	0,6 [0,59 - 0,65]
Surface aortique^1^	0,6 ± 0,2
DTD VG (mm)^1^	62 ± 7,4
DTS VG (mm)^1^	49 ± 8
FE^1^(%)	32,3 ± 5,3
Gradient moyen trans-aortique^1^ (mmHg)	51,6 ± 19
PAPS^1^ (mmHg)	48,7 ± 23,5
Euroscore logistique^1^	12,2 ± 8,7

1 : exprimée en moyenne ± écart-type;

2 : exprimée en médiane [écart interquartile];

3 : exprimée en effectif (pourcentage); ICC: insuffisance cardiaque congestive; HTA: hypertension artérielle; IR: insuffisance rénale; AVCI: accident vasculaire cérébrale ischémique; DTD VG: diamètre télédiastolique du ventricule gauche; DTS VG: diamètre télésystolique du ventricule gauche; FE: fraction d’éjection du ventricule gauche; PAPS: pression artérielle pulmonaire systolique.

**Tableau 2:** Données opératoires et résultats postopératoires précoces

Variables	n = 46
Temps de Clampage Aortique^1^ (mn)	70,5 ± 21,7
Temps de CEC^2^ (mn)	91,5 [80,7; 129,5]
Diamètre de prothèse^1^ (mm)	21,8 ± 1,6
Usage de drogue inotrope^3^	42 (91,3%)
Délai d’extubation^2^ (h)	8,5 [7 ; 10]
Durée de séjour en réanimation^2^ (h)	48 [48; 72]
DTD VG post-opératoire^1^ (mm)	60,3 ± 8
DTS VG post-opératoire^1^(mm)	46,4 ± 8,4
FE post-opératoire^1^ (%)	37,8 ± 10
Gradient moyen^1^ (mmHg)	12,4 ± 3,7
**Complications**	
-Saignement^1^ (ml)	453,5 ± 265
-Bas débit^3^	16 (35%)
-IRA	2 (4,4%)
-BAV III^3^	1 (2,2%)
-Reprise^3^	1 (2,2%)
Mortalité^3^	7 (15,2%)

1 : exprimée en moyenne ± écart-type ;

2 : exprimée en médiane [écart interquartile] ;

3 : exprimée en effectif (pourcentage) ; CEC : circulation extracorporelle ; DTD VG: diamètre télédiastolique du ventricule gauche ; DTS VG: diamètre télésystolique du ventricule gauche ; FE: fraction d’éjection du ventricule gauche ; IRA: insuffisance rénale aigue ; BAV III:bloc auriculoventriculaire 3eme degré.

**Tableaux 3:** Etude des facteurs de risque de mortalité hospitalière par analyse de la régression logistique (en uni et multivariée)

Variables	Analyse univariée			Analyse multivariée		
	0R Brute	IC	P Value	0R Ajusté	IC	P Value
Age	1,02	[0,95- 1,10]	0,51			
sexe	0,65	[0,68 ; 6,16]	0,70			
**IR**	11,66	[1,90- 72]	**0,008^+^**	11,94	[2,65-72,22]	**0,03^+^**
**ICC**	24,66	[3,13- 194,53]	**<0,001^+^**	25,33	[3,43- 194,74]	**0,009^+^**
**NYHA**	8,20	[1,60- 42,30]	**0,01^+^**	10,60	[0,20- 577]	0,25
ICT	16875	[0,001- 2 10^11^]	0,24			
Nature de la valvulopathie	1,42	[0,30- 7,22]	0,67			
**FE préopératoire**	0,86	[0,77- 0,96]	**0,01^+^**	1,00	[0,80- 1,23]	0,99
DTD VG préopératoire	1,00	[0,90- 1,12]	0,93			
DTS VGpréopératoire	1,05	[0,95- 1,16]	0,30			
Gradient transvalvulaire moyen	1,00	[0,96- 1,04]	1			
Surface valvulaire Aortique	0,45	[0,005- 39,30]	0,72			
PAPS	1,00	[0,98- 1,05]	0,46			
Temps de Clampage aortique	1,00	[0,96- 1,04]	0,94			
**Temps de CEC**	1,02	[1,00- 1,04]	**0,02^+^**	1,02	[0,99- 1,05]	0,16
Saignement post- opératoire	1,00	[0,99- 1,00]	0,41			

IR: insuffisance rénale; ICC: insuffisance cardiaque congestive; FE: fraction d’éjection du ventricule gauche; DTD VG: diamètre télédiastolique du ventricule gauche; DTS VG: diamètre télésystolique du ventricule gauche; PAPS: pression artérielle pulmonaire systolique.

+ : P significatif pour un risque α fixé à 0,05

**Figure 1:**
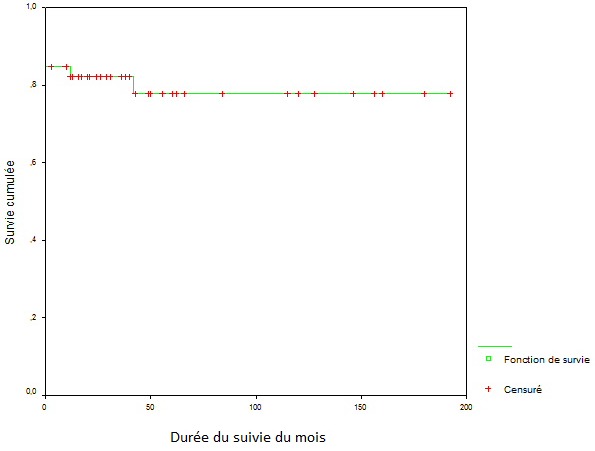
Survie cumulée analysée par la méthode de Kaplan-Meier

Les trente neuf patients survivants ont été bien suivis. La durée moyenne de suivi a été de 59,6± 21mois. La mortalité tardive était de 5% (un patient est décédé après un accident vasculaire cérébral hémorragique et un deuxième patient dans les suites d'un lymphome malin non hodgkinien) (Figure 1). Le statut fonctionnel a été nettement amélioré (81% des patient était en classe I de La NYHA), et seuls 35% des patients ont nécessité un support digitalo-diurétique au long cours (Tableau 4). La comparaison de la FE entre la période préopératoire, postopératoire précoce et postopératoire tardive a révèle une différence statistiquement significative (p< 0,001). En effet la FE a augmentée, en moyenne, de 5,5 unités en période postopératoire précoce et de 18 unités en postopératoire tardive. En fin, nous avons constaté en postopératoire précoce, une régression moyenne du DTD et DTS, successivement, de 2 et 2,5 mm. De même, en postopératoire tardive le DTD et DTS moyens ont diminué respectivement de 8 et 9 mm (Tableau 5).

**Tableau 4:** Résultats postopératoires tardifs

Variables	n = 46
Durée de suivi ^1^(mois)	59,6± 21
NYHA^3^	
-I	30 (81%)
-II	20 (19%)
Support digitalo-diurétique^3^	13 (35%)
Complications tardives^3^	
-AVC	2 (4,3%)
-Poussée d’insuffisance cardiaque gauche	2 (4,3%)
DTD VG (mm)^1^	54,2 ± 8
DTS VG (mm)^1^	40 ± 7,5
FE^1^ (%)	50,3 ± 10
Mortalité tardive^3^	2 (5 %)

1 : exprimée en moyenne ± écart-type;

2 : exprimée en médiane [écart interquartile];

3 3: exprimée en effectif (pourcentage); AVC: accident vasculaire cérébrale; DTD VG:diamètre télédiastolique du ventricule gauche; DTS VG:diamètre télésystolique du ventricule gauche; FE: fraction d’éjection du ventricule gauche.

**Tableau 5:** Comparaison de la fraction d’éjection, des diamètres télédiastolique et télésystolique du ventricule gauche en préopératoire, postopératoire précoce et postopératoire tardif

Variables	Préopératoire (G1)	Postopératoireprécoce(G2)	Postopératoire tardif (G3)	P Value
**FE**	32 ± 8	37,8 ± 10	50,3 ± 10	G2 Vs G1 p < 0,001^+^
				G3 Vs G2 p < 0,001^+^
				G3 Vs G1 p < 0,001^+^
**DTD VG**	62 ± 7,4	60,3 ± 8	54,2 ± 8	G2 Vs G1 p = 0,001^+^
				G3 Vs G2 p < 0,001^+^
				G3 Vs G1 p < 0,001^+^
**DTS VG**	49 ± 8	46,4 ± 8,4	40 ± 7,5	G2 Vs G1 p < 0,001^+^
				G3 Vs G2 p < 0,001^+^
				G3 Vs G1 p < 0,001^+^

+ Significatif pour un p < 0, 05.

DTS VG: diamètre télésystolique du ventricule gauche; FE: fraction d’éjection du ventricule gauche; DTD VG: diamètre télédiastolique du ventricule gauche.

## Discussion

Le RAo serré avec dysfonctions VG représente 5-15% le l'ensemble des RAo [1, 2, 5,6]. Le pronostic spontané, en l'absence de traitement chirurgical, est sombre avec taux de survie à un an ne dépassant pas 24 % [7]. Actuellement, en dehors de la transplantation cardiaque dont on connait les contraintes, la seule thérapie efficace est la levée de l´obstruction mécanique par remplacement valvulaire aortique (RVA) ou par implantation de bioprothèse par voie percutanée (TAVI) comme option thérapeutique [8]. Le bénéfice du RVA dans RAo avec dysfonction ventriculaire gauche a été bien démontré en 1978 par Smith et al dans une série de 19 patients [9] , puis confirmé dans une plus grande série de 154 patients avec gradient transvalvulaire élevé [10] . Cependant le risque opératoire demeure important. Ainsi, nous avons observé un taux de mortalité hospitalière de 15,20 % lié essentiellement au bas débit. Ce taux rejoint la marge de 8 à 21% rapporté dans la littérature [2,4]. En effet, le pronostic post-chirurgical des patients ayant une dysfonction VG sévère semble meilleur par rapport aux patients traités médicalement. Mieux encore, la survie se voit accompagnée d´une amélioration du statut fonctionnel [2,11]. Plusieurs facteurs de risques de mortalité hospitalière ont été rapportés notamment : l'âge, le sexe féminin, la classe III-IV de la NYHA, l'insuffisance rénale, l'insuffisance cardiaque globale, le DTS du VG>54mm, l'hypertension artérielle pulmonaire sévère, l'absence de réserve contractile (RC) et le faible gradient valvulaire transaortique [4, 5,12-14]. Cependant la plupart des séries publiées sont hétérogènes incluant des patients avec d'autres lésions associées, en particuliers, des lésions coronaires ou valvulaires autres que le rétrécissement aortique. Le caractère péjoratif de l'association cardiopathie ischémique et sténose aortique serré a été bien démontré par Powell et al qui ont rapporté un taux de mortalité opératoire de 45% sur une série de patients avec d'antécédents d'infarctus du myocarde [15]. De même la présence de lésions valvulaire, notamment une insuffisance mitrale modérée, est rapportée comme liée à un risque postopératoire élevé [12,16].

Dans le but de supprimer le biais de l'effet des lésions associées sur le pronostic, notre étude s'est intéressée au RAo serré isolé avec dysfonction ventriculaire gauche sévère. Elle a révélé comme facteurs de risque de mortalité hospitalière : la classe fonctionnelle III et IV, l'insuffisance rénale, l'insuffisance cardiaque congestive, la FE effondrée, et le temps de CEC allongé. Et en ajustant sur ces derniers facteurs, une à une, l'analyse multivariée a permis de déceler l'insuffisance rénale et l'insuffisance cardiaque congestive comme les deux seuls facteurs statistiquement liés au risque de mortalité hospitalière. Dans une étude multicentrique, Clavel et al ont montré que les patients ayant un faible gradient sont une population à risque élevé avec une mortalité opératoire de 18%, ce risque était encore plus élevé lorsque le gradient transvalvulaire était ≤20 mmhg [6,17]. Notre étude qui n'a pas constaté de hausse de mortalité hospitalière relative à un bas gradient, concorde avec les résultats de Borowski qui a rapporté un taux de mortalité postopératoire presque identique entre le groupe de bas gradient et celui de haut gradient [18]. Depuis 2012 nous avons commencé à étudier la réserve contractile (RC) du VG par échocardiographie à la dobutamine chez les patients ayant un bas débit-bas gradient, c'est ainsi que trois de nos patients en ont bénéficié. Ils possédaient tous des réserves contractiles. La réserve contractile du VG est un facteur déterminant du risque opératoire. Tribouilloy a retrouvé un taux de mortalité opératoire de 32% chez les patients qui n'avaient pas de RC contre 5% chez ceux qui en avaient [14, 19, 20]. De même Monin et ses collègues ont montré que les patients ayant une RC avaient un meilleur pronostic que ceux sans RC, cependant Les deux groupes de patients avaient une meilleure espérance de vie par rapport aux malades traités médicalement [21]. Ainsi, même si l'absence de RC est corrélée à une forte mortalité postopératoire, elle ne doit pas constituer, à elle seule, une contrindication à la chirurgie d'autant plus que les chances de récupération myocardique après la chirurgie ne sont pas exclues [22].

À long terme le taux de survie calculée de nos patients était de 80,40% après un suivi moyen de 59,6± 21mois. Plusieurs facteurs de risque sont décrits comme étant liés au risque de mortalité tardive notamment : L'HTAP sévère, la classe III ou IV de la NYHA en préopératoire, L'usage de fortes doses de diurétique préopératoire, le sexe masculin, et l´âge avancé [23]. Nous n'avons pas retrouvé ses facteurs comme liées au risque de mortalité tardive puisque nos 2 décès tardifs étaient de causes non cardiaques. Morris et al ont rapporté que 72 % des patients qui ont eu une amélioration de la fraction d´éjection en postopératoire avaient une meilleure courbe survie [24]. Vaquette et al ont conclu que les patients dont l'amélioration précoce de la FE était de plus de10 unités avaient une meilleure survie à long terme que les patients dont la FE a augmenté de moins de 10 unités [25]. Notre étude démontre qu'une augmentation de la FE moyenne de 5,5 unités en postopératoire immédiate était associée à une amélioration du taux de survie. Nous avons constaté une amélioration de la classe fonctionnelle de la NYHA (81% de nos patients étaient en classe I de La NYHA). Cette amélioration fonctionnelle était bien corrélée au degré de récupération de la fonction ventriculaire gauche. Il a été rapporté qu'il existe une relation étroite entre la taille du VG en préopératoire et la possibilité de récupération ventriculaire. Ainsi, Une et al ont rapporté que les patients dont le DTD préopératoire du VG était ≤55 mm avaient plus de chance d'améliorer leur fonction ventriculaire gauche après RVA [26]. Pour des diamètres préopératoires moyens de 62 ± 7,4 mm en télédiastole et de 49 ± 8 mm en télésystole, nous avons observé une régression tardive de ces deux diamètres de 8 et 9 mm respectivement avec une augmentation de la FE de18 unités. En effet, lorsque la dysfonction ventriculaire est liée à augmentation de la postcharge, le remplacement de la valve permet une augmentation de FE et une amélioration fonctionnelle tant qu'il n´y a pas d´autres causes de cette dysfonction ventriculaire (infarctus du myocarde, valvulopathie concomitante, etc.) [12,27]. Cependant, lorsque la fibrose myocardique s'installe et devient est irréversible, le risque de mortalité postopératoire augmente et les chances de récupération de la fonction ventriculaire gauche sont moins probables [6,28]. Ainsi, l'absence de récupération de la fonction ventriculaire gauche peut être attribuable, dans de nombreux cas, aux lésions de fibrose myocardique qui entravent la contractilité myocardique. D'où l'intérêt de l'étude de la réserve contractile par échocardiographie sous dobutamine qui permet, en plus de la stratification du risque opératoire, de prédire l'évolution tardive [28,29].

## Conclusion

Les résultats du remplacement valvulaire aortique sur rétrécissement aortique serré avec dysfonction sévère du ventricule gauche sont satisfaisants. L'insuffisance cardiaque congestive et l'insuffisance rénale préopératoires constituent les principaux facteurs de risque de mortalité hospitalière. L'évolution tardive est marquée par une régression des diamètres télésystolique et télédiastolique du ventricule gauche avec amélioration de la FE et du statut fonctionnel. Limites de l'étude: la première limite de l´étude est inhérente à sa nature rétrospective. La petite taille de l´échantillon pourrait diminuer le poids des résultats. En outre, le jeune âge de nos patients et l'hétérogénéité des étiologies pourraient influencer les résultats. Toutes nos conclusions devraient être considérées dans ce contexte.

### Etat des connaissances actuelles sur le sujet

- Le RAo serré avec dysfonctions VG sévère représente 5-15% le l'ensemble des Rao;
- Le pronostic post-chirurgical semble meilleur par rapport aux patients traités médicalement;
- Plusieurs facteurs de risque de mortalité postopératoires sont différemment rapportés.

### Contribution de notre étude à la connaissance

- Nôtre étude s'est intéressée qu'au RAo serré isolé avec dysfonction ventriculaire gauche sévère afin d'éliminer les effets probables des lésions associées sur les résultats postopératoires et sur le risque de mortalité hospitalière;
- L'insuffisance cardiaque globale et l'insuffisance rénale préopératoires constituent les principaux facteurs de risque de mortalité postopératoire;
- L'évolution tardive est marquée par une régression des diamètres télésystolique et télédiastolique du ventricule gauche avec amélioration de la FE et du statut fonctionnelle.

## Conflits d’intérêts

Les auteurs ne déclarent aucun conflits d'intérêts.
